# Bisphosphonate-related osteonecrosis of the jaws (Bronj)

**DOI:** 10.4317/medoral.18076

**Published:** 2013-05-31

**Authors:** Francesco Beninati, Riccardo Pruneti, Giuseppe Ficarra

**Affiliations:** 1 Research Fellow, Reference Center for the Study of Oral Diseases, University of Florence, Italy; 2 Director, Reference Center for the Study of Oral Diseases, University of Florence, Italy

## Abstract

Bisphosphonate-related osteonecrosis of the jaws (BRONJ) is an extremely therapy resistant osteomyelitis-like disease exclusively involving the jaw bones of patients in treatment with bisphosphonates (BPs). Objectives: The aim of this study was to evaluate the radiological and clinical findings and management of 51 patients with BRONJ diagnosed from 2004 to 2009 in our Reference Center. Study Design: A prospective study was performed. The patients were examined every 2-6 months, depending on their clinical conditions. Outcome variables were the resolution of symptoms, persistence of bone exposure and /or fistula and the status of the lesional mucosa. Results: The higher prevalence of the disease was noted in 2006 and 2007 and at the time of diagnosis 90% of patients had been treated with iv BPs. The main precipitating event leading to BRONJ was an invasive dental procedure in 61% of patients while no traumatic event could be identified in 16% of patients. The median time of follow-up was 19 months (range: 2-57), during which 31% of patients healed and 39% succumbed. In 78% of patients the therapy was medical, in 16% it consisted in surgical deep curettage and only in 6% it was necessary to perform an osteotomy to avoid a mandibular pathological fracture. All the patients in treatment with oral BPs healed from BRONJ with a median time of conservative treatment of 19 months. Conclusions: Prevention has lead to a progressive reduction in the prevalence of BRONJ. In our experience medical treatment is often sufficient to keep the disease under control and to lead to the healing of the lesions by spontaneous loss of the sequestrum. This approach seems to be very effective in patients who were in treatment with oral Bps preparations; BRONJ seems to have a more benign clinical behaviour in these patients.

** Key words:**Bisphosphonates, osteonecrosis, treatment, follow-up.

## Introduction

Bisphosphonate-related osteonecrosis of the jaws (BRONJ) is an extremely therapy resistant osteomyelitis-like disease exclusively involving the jaw bones of patients in treatment with bisphosphonates (BPs). The potent nitrogen-containing BPs (e.g. pamidronate, zoledronic acid, alendronate, risedronate and ibandronate), predominantly when administered intravenously (iv), have been more often associated with this disease. The incidence of BRONJ remains undefined and it ranges from 0.8 to 12% for i.v. preparations; the incidence for oral preparations ranges from 0,01 to 0,06 % and after oral invasive treatments this rate increases from 0,07 to 0,34% (1-7).

BRONJ is more often localized in the mandible than in the maxilla (2:1 ratio), it is usually caused by a dental surgical procedure (60-70% of cases) or a prosthetic trauma and it is more rarely spontaneous (1-7).

The mechanism of action of bisphosphonates is not yet well understood, but it essentially involves a powerful inhibition of bone resorption as a result of the reduction of osteoclast activity; as far as nitrogen-containing BPs are concerned they are also thought to have antiangiogenic effects (8).

The first cases of BRONJ were observed in 2003 and all the initial observations have pointed on the potential role of the intravenously administered bisphosphonates (9-14). Additionally, BRONJ has been reported in a small number of patients who had received oral non-nitrogen or oral nitrogen-containing bisphosphonates both in cancerous and non-cancerous conditions.

The aim of this study is to present the clinical and radiological features and the follow-up data of 51 patients affected by BRONJ and primarily treated with a conservative non- surgical approach.

## Material and Methods

From 2004 to 2009 51 patients with BRONJ were observed and prospectively followed at the Reference Center for the Study of Oral Diseases, Florence, Italy. The diagnosis was performed basing on clinical and radiographic features and biopsy was carried out only when necessary to exclude other diseases. To stage the lesions were used the most authoritative criteria for BRONJ put forth by the AAOMS (1,2). In all cases, a panorex or a computerized tomography of the jaws was performed.

In keeping with current published guidelines, all the patients were initially treated with a conservative approach, using nonalco-hol-containing chlorhexidine 0.12% mouth rinse, local irrigation with povidone-iodine, superficial curettage or conservative de-bridement of bone sequestra, intermittent oral antibiotics and pharmacological pain control as clinically required (1,2). Conservative debridement consisted in a non-aggressive, superficial removal of bone sequestra while the goal of superficial curettage was to eliminate dead bone, foreign material and plaque without exposure of additional bone. The dosage of amoxicillin/clavulanate potassium was 1000 mg tabletes every 12h for 15-20 days associated, in resistant cases, to 250 mg metronidazole every 8 h for 5 days. In penicillin allergic patients 300 mg clindamycin was administered three times a day. Pain control was obtained by oral administration of nonsteroidal anti-inflammatory drugs (nimesulide or ibuprofen) preferably, or opioids (tramadol hydrochloride). Surgical treatments (flap osteotomy), were performed only when we were unable to control the necrosis of the bone, to avoid severe complications. Hyperbaric oxygen therapy was not used. The continuation of bisphosphonate treatment after BRONJ was diagnosis was left to the discretion of the treating physician. The patients were then examined every 2-6 months, depending on their clinical conditions.

## Results

From 2004 to 2009 51 cases of BRONJ were diagnosed; the higher prevalence of the disease was noted in 2006 and 2007, while the lower one was during 2009. The median age of patients was 68 years and the sex ratio was F:M = 3:2 (30 females and 21 males). At the time of diagnosis, 46 patients (90% of cases) had been treated with iv BPs for oncological reasons for a median of 28 months (range 3-120) while 5 patients (10%) affected by non-oncological diseases were treated with oral BPs for a median of 48 months (range 24-84) ( Table 1). Multiple myeloma was the most common oncological disease (31% of cases) followed by metastases from breast cancer (27% of cases). Thirty-four patients (67%) underwent cancer treatments during the year preceding the onset of BRONJ. The oncological treatment modalities in the studied group varied widely and included chemotherapeutic agents (alkalyting agents, antimetabolites), hormonal treatments (antiandrogens, antiestrogens, aromatase inhibitors) and inhibitors of angiogenesis (thalidomide).

**Table 1:**
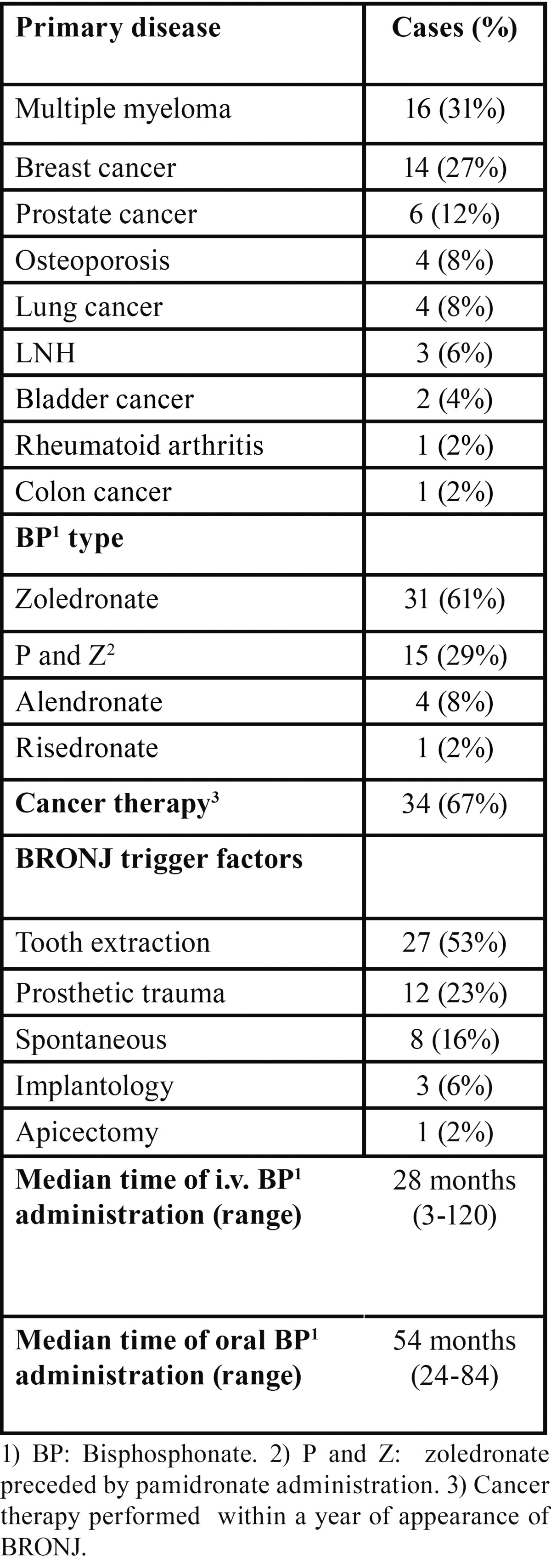
Epidemiological data of 51 BRONJ patients.

The precipitating event leading to BRONJ was an invasive dental procedure in 31 patients (61%) that was dental extraction in 27 of them (53% of all patients). A prosthetic trauma was found in 12 patients (23%) while no traumatic event could be identified in 8 patients (16%). The mandible was affected in 31 patients (61%), the maxilla in 13 patients (25%), while both jaws were affected in 7 patients (14%). The lesions observed were 65 in all, 66% of which were mandibular. Using the proposed staging criteria for BRONJ, at diagnosis 3 patients (5%) presented with stage 1 disease, 46 patients (90%) with stage 2, and 2 patients with stage 3 (5%). The bisphosphonate treatment was stopped by the treating physician in all patients. At diagnosis 20 patients felt pain (39%), 4 patients (8%) presented trismus and one patient (2%) paresthesia ( Table 2). The lesions most frequently showed as an area of bone exposure (85% of lesions) and more rarely a fistula (15% of lesions).

**Table 2:**
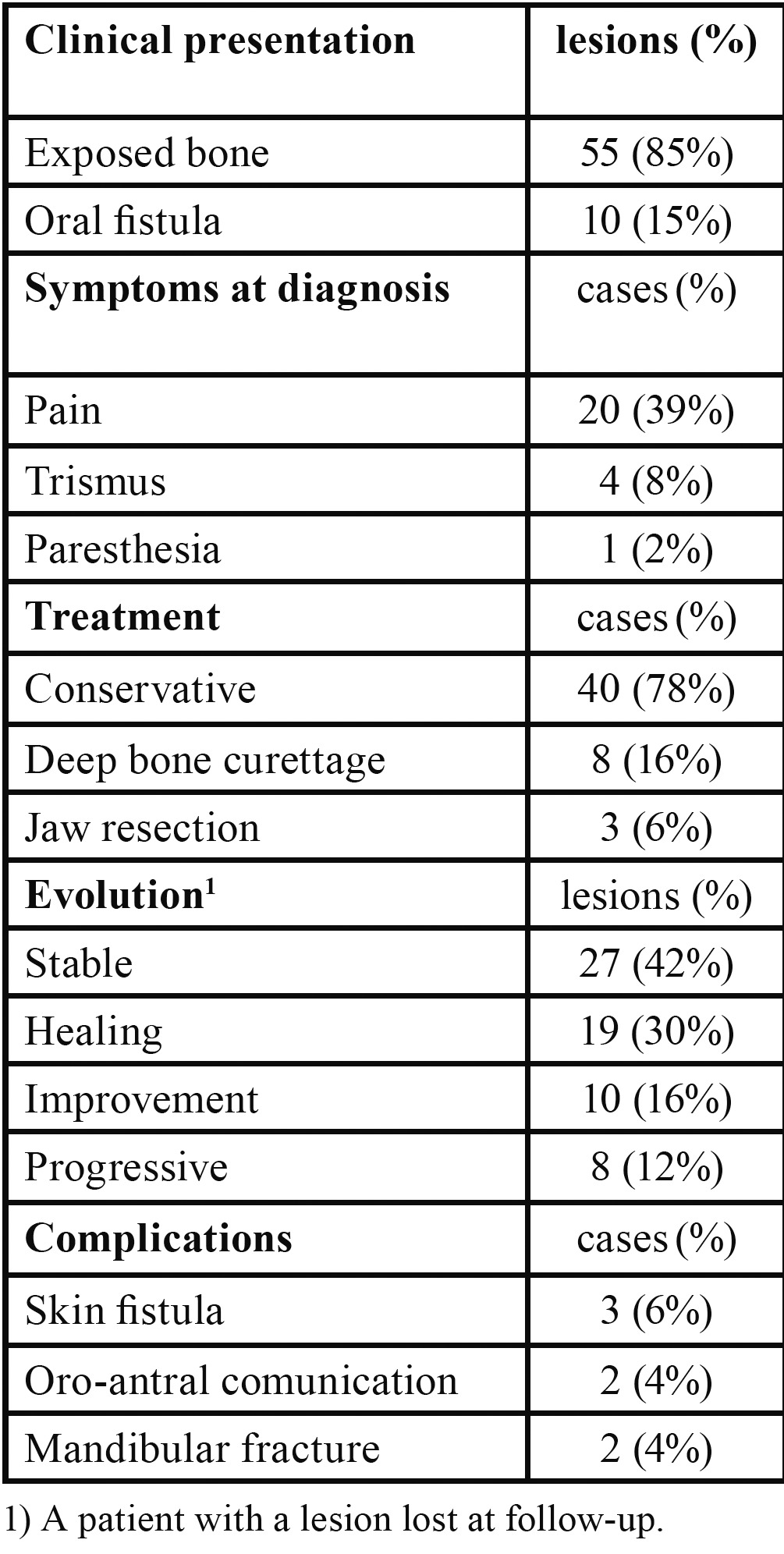
Clinical aspects (65 oral lesions of 51 BRONJ patients).

The median time of follow-up was 19 months (range 2-57), during which 16 patients (31%) healed while 20 patients (39%) succumbed from complications related to their malignancy; only a fatality could be attributed directly to BRONJ complications (mandibular fracture and consequent surgical treatment). Only a patient was lost to follow-up.

In 40 patients (78%) the therapy was not surgical, while in 8 patients (16%) it consisted in surgical deep curettage; only in 3 patients (6%) it was necessary to perform an osteotomy to avoid a mandibular pathological fracture. Of the 64 lesions followed (a patient with a lesion was lost at follow-up), 42% remained stable (27 lesions), 16% improved (10 lesions), 30% healed (19 lesions) and 12% worsened (8 lesions).

**Figure 1:**
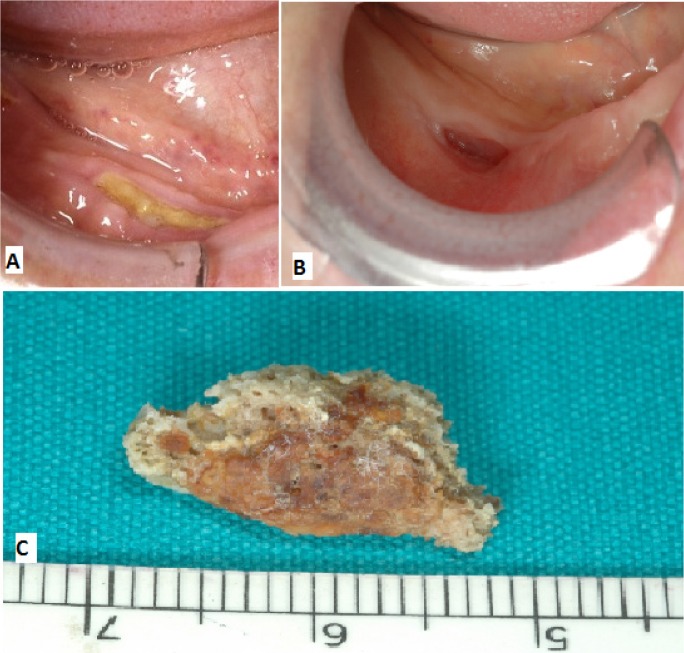
A) Healing after conservative treatment: clinical features at diagnosis. B) Spontaneous healing following loss of bone sequestrum 18 months after diagnosis. C) Sequestrum.

**Figure 2:**
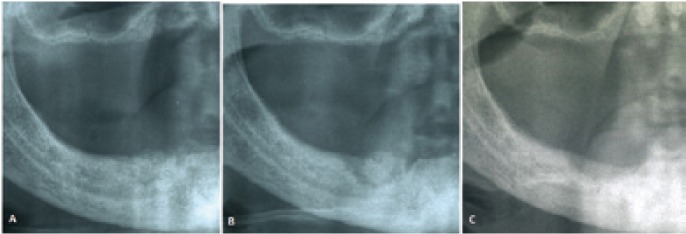
A) Healing after conservative treatment: radiological features at diagnosis. B) Radiological features after 12 mounths. C) Radiological features after 18 mounths (healing).

14 of 19 healing lesions were exclusively treated with a conservative approach and healed after spontaneous sequestrum loss ( Table 3), (Figs. 1,2). Necrotic bone loss resulted in a fistula which healed after local irrigation with saline and povidone-iodine in only 2 cases. The remaining 5 healing lesions were treated surgically, but primary healing was observed in 3 cases only ( 2 cases treated with osteotomy and 1 case with deep curettage). All the patients with BP oral administration healed and were exclusively treated with conservative treatment.

**Table 3:**
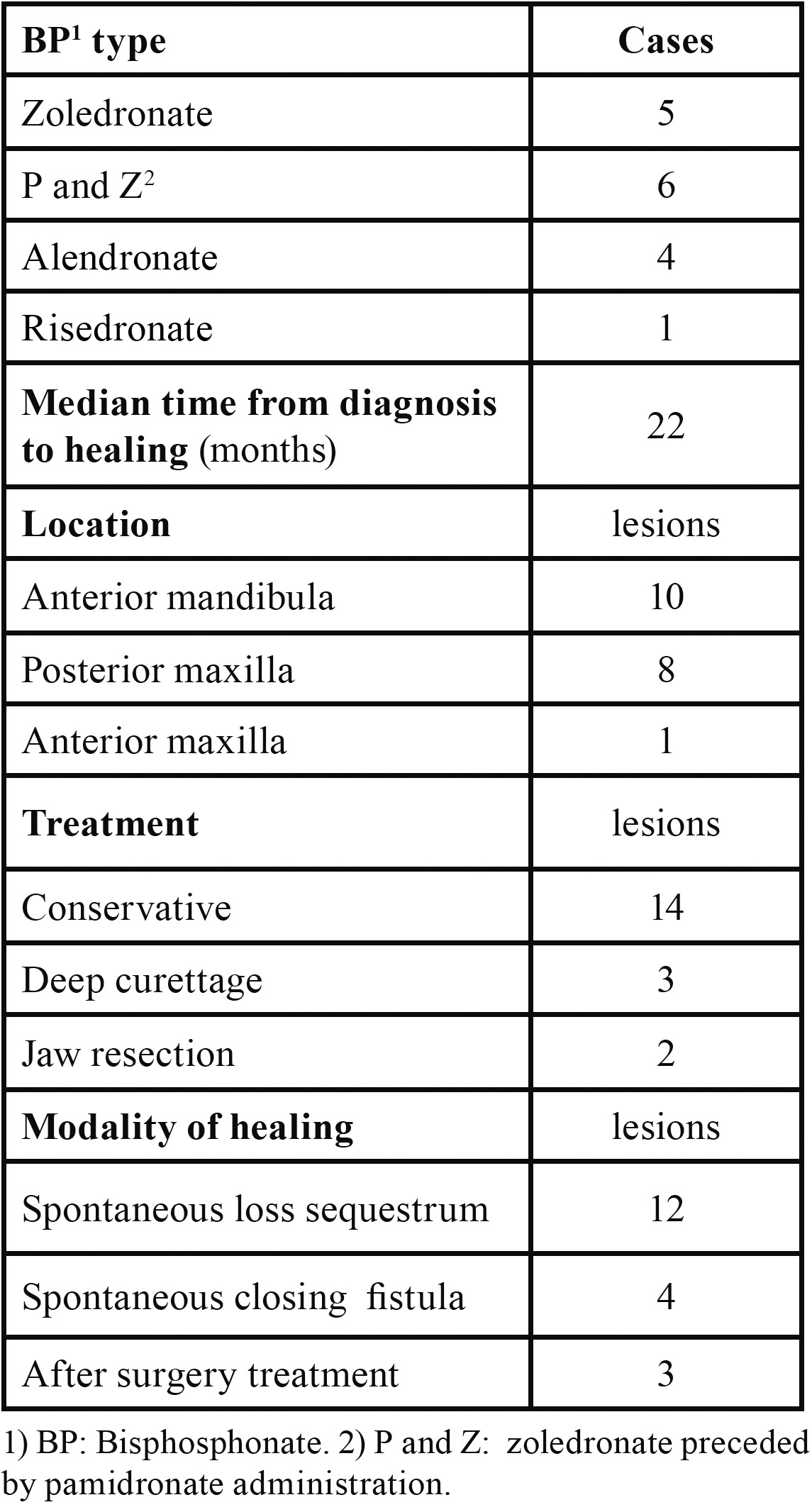
Clinical aspects of the16 healed patients (19 lesions).

**Figure 3:**
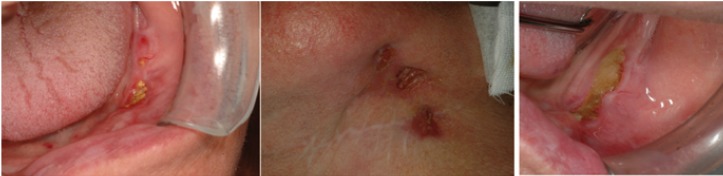
Three years follow-up in a stage 3 patient. Oral lesion and skin fistula at diagnosis and oral lesion after a year.

**Figure 4:**
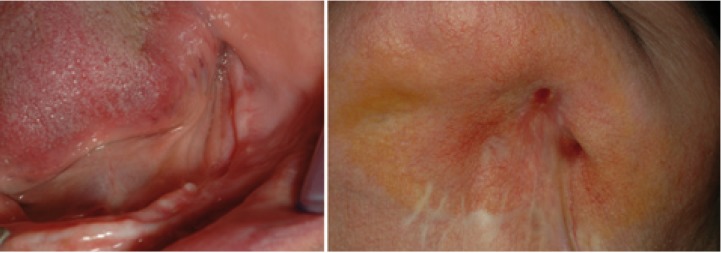
Three years follow-up in a stage 3 patient. Oral lesion and skin fistula after three years.

The most common complication was the appearance of a skin fistula (6% of cases) while oro-antral communications rarely occurred (4% of cases) as well as pathological mandibular fractures (4% of cases) (Figs. 3,4).

At diagnosis the most common radiographic features of the necrotic bone were worm-eaten appearance (32% of lesions) followed by superficial osteolysis (24% of lesions), while osteolysis was limited to cortical bone without bone marrow involvement; these radiological aspects corresponded to a more favourable clinical condition. In some cases it was not possible to identify any radiological lesion (12% of lesions) despite the presence of clinical bone exposure ( Table 4).

**Table 4:**
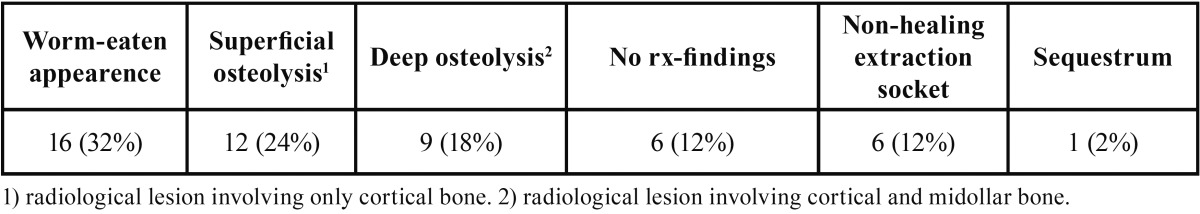
Radiological findings at diagnosis (50 lesions).

## Discussion

Two classes of BPs are in clinical use today: a less potent group of non-nitrogen containing molecules (etidronate and clodronate) and a newer class of very potent agents that contain a nitrogen moiety (pamidronate, zoledronic acid, alendronate, risedronate, ibandronate and others). Besides bone effects the nitrogen-BPs have antitumoral and antiangiogenesis properties, which make them very important drugs in the treatment of primary and secondary bone oncologic disease (15).

Various hypotheses have been proposed about the aetiology of BRONJ but fundamental questions remain unanswered. Probably the aetiology is multifactorial, where the disruption of bone remodeling, local trauma, pre-existing dental disease, concomitant cancer therapies, reduced angiogenesis and superinfection lead to a disrupted bone microenvironment prone to osteonecrosis. The exact role these different factors play in the pathogenetic process remains to be esta-blished (2,16,17).

Only the mandible and maxilla appear to be susceptible, highlighting their unique nature compared with other parts of the skeleton. The jaws are the only bones in the human body that are in frequent contact with the outside world and are subject to repeated microtrauma through the presence of teeth and the forces of mastication; moreover the turnover of alveolar bone is 10-fold greater than in the long bones. While BPs can decrease this turnover, the alveolar remodeling still remained higher compared with the long bones. Local trauma caused by tooth extractions, local surgery or ill-fitting dentures are the foremost important risk factors, being consistently reported throughout the literature (2).

Apparently there is no clear evidence that the association of BPs and a particular cytotoxic agent or class of chemotherapic agents increases the risk of BRONJ onset. Otherwise, many cases have been reported with alkylators alone (2).

According to literature BRONJ mainly affects people over 60 years of age and females more often than males; diseases that more frequently underlie BRONJ are multiple myeloma first of all , then, in order of frequency, breast cancer , prostate cancer and osteoporosis (2-7).

Approximately 90% of reported BRONJ cases received zoledronic or pamidronic acid while the remainder of patients were treated with oral bisphosphonates such as alendronate or risedronate (2-7).

An always increasing number of cases concerns the use of intravenously administered ibandronate, a newer agent licensed in the US for the treatment of osteoporosis and also available as an oral formulation.

BRONJ affects the mandible more often than the maxilla, while both jaws involvment is rare.

Over 90% of cases of BRONJ follow a trigger event such as a surgical or a prosthetic trauma, while a spontaneous onset is rare (2-7). All of these epidemiological data are in accordance with those we found.

Diagnosis of BRONJ should be mainly based on clinical and radiographic criteria. Tissue biopsy is not always necessary and should be performed only if metastatic disease is suspected.

American Association of Oral and Maxillofacial Surgeons position paper guidelines define the disorder as the persistence of exposed bone in the oral cavity in patients treated with BPs, after adequate management for 8 weeks, in the absence of local metastatic disease and without previous radiation therapy to the affected area (1,2).

The clinical aspects and behaviour of BRONJ show a striking resemblance to osteoradionecrosis with exposed bone and sequestration non-responsive to conventional surgical management. BRONJ may be asymptomatic for long time or may result in pain or exposed maxillary or mandibular bone. Typical signs and symptoms are pain, soft-tissue swelling and infection, loosening of teeth and draining fistula. Patients may have other symptoms, which are bred by the infection, such as trismus, halitosis and recurrent abscesses, sinusitis with or without oro-antral fistula (2,18).

Staging criteria for BRONJ have been proposed, basing on the model of cancer tumour staging. The most authoritative to date are those put forth by the AAOMS (1,2). The ASBMR (American Society for Bone and Mineral Research) task force has recently recommended the development of a more comprehensive staging and grading system that is based not only on physical examinations and symptoms but on imaging and other parameters as well, in order to better define severity and assess treatment response (19).

Complications that may be observed in association to BRONJ are, in order of frequency: skin fistula, maxillary sinusitis with oro-antral and/or oro-nasal communications and pathologic fracture of the mandible.

A panorex is commonly used for the diagnosis of BRONJ, but tomographic images are needed to establish the real extent of bone necrosis and for a more accurate diagnosis of the complications (20,21).

Magnetic resonance imaging, cone-beam computerized tomography and nuclear bone scan (PET-TC) have been proposed to better identify the osteonecrotic area and make an earlier diagnosis (21,22).

Radiographic alterations are not evident until there is significant bone involvement. Late radiographic changes may mimic classic periapical inflammatory lesions or osteomyelitis. Other radiographic findings include non-healing extraction site, widening of the periodontal ligament space and osteosclerotic lamina dura (21).

In our experience the most frequent radiographic aspects at the time of diagnosis are worm-eaten like lesions (32% of lesions) followed by superficial osteolysis (24% of lesions).

Currently there are no effective treatments for BRONJ even if a large variety of treatment modalities have been reported, including conservative medical management, various types of surgery, hyperbaric oxygen, ozone therapy and laser therapy (2,21,23). The AAOMS has proposed a staging system in order to select the best treatment strategy for a given patient. Patients with stage 1 and 2 BRONJ should be treated using a conservative approach with the goal of preventing progression of lesions and limiting complications related to chronic infection (2). To achieve these goals, conservative debridement of bone sequestra, local irrigation with povidone-iodine and daily rinsing with 0,12% chlorhexidine mouthwash, antibiotic therapy and pain control should be considered. Surgical debridement has been variably effective in eradicating the necrotic bone. The goal of surgery should be to elimi-nate dead bone which acts as foreign material; thus necrotic areas that are a constant source of soft tissue irritation should be removed or recontoured without exposure of additional bone. Recent findings have further confirmed that non-surgical medical management of BRONJ, consisting in long-term administration of antibiotics, is very effective in controlling the progression of the disease. In case of stage 3 patients with pathologic mandibular fractures segmental resection and reconstruction are required.

In our experience conservative non surgical management is a valid approach to BRONJ considering the poor health of BRONJ affected people; in our study conservative medical management made 42% of BRONJ lesions become stable and 30% of them heal spontaneously; we observed the healing of the lesions in all the patients with BP oral administration.

Five of our patients were destined to segmental resection of the mandible; one of these died from surgical complication, 2 of these healed and the other 2 ones refused surgery and have been under conservative treatment without pathologic fracture of the mandible occurrence until now.

It is very important that preventive measures are always taken in order to subvert the risk of developing this severe complication. These include careful dental examination and preventive extractions of candidate teeth with enough time allowed for healing in advance of the start of BPs treatment. Moreover, all patients taking BPs have to be informed of the benefits and risks of treatment and encouraged to maintain good oral hygiene (including regular dental visits) (2).

## Conclusions

Prevention has lead to a progressive reduction in the prevalence of BRONJ. Since BRONJ patients’ poor health and uncertainty of surgical outcomes, conservative management is to be preferred. In our experience medical treatment is often sufficient to keep the disease under control and to lead to the healing of the lesions by spontaneous loss of the sequestrum.

This approach seems to be very effective in patients who were treated with oral BP preparations; BRONJ seems to have a more benign clinical behaviour in these patients.
